# 599 To Titrate or Not to Titrate, That Is the Burning Question: Enoxaparin and VTE Prophylaxis

**DOI:** 10.1093/jbcr/irae036.233

**Published:** 2024-04-17

**Authors:** Kevin Vega, Morgan Palumbo, Ke Cheng, Jeffrey Anderson

**Affiliations:** Temple University Hospital, Philadelphia, PA; Temple University, PHILADELPHIA, PA; Temple University, Wallingford, PA; Temple University Hospital, Philadelphia, PA; Temple University, PHILADELPHIA, PA; Temple University, Wallingford, PA; Temple University Hospital, Philadelphia, PA; Temple University, PHILADELPHIA, PA; Temple University, Wallingford, PA; Temple University Hospital, Philadelphia, PA; Temple University, PHILADELPHIA, PA; Temple University, Wallingford, PA

## Abstract

**Introduction:**

Patients who sustain burn injury undergo metabolic and physiologic changes that place them at elevated risk of developing venous thromboembolism (VTE). Although the benefit and safety of enoxaparin titration for prevention of VTE in trauma patients is well established, there is a lack of evidence regarding its applicability for burn injured patients. In this study, we sought to determine the safety and applicability of enoxaparin titration for burn injured patients admitted for treatment of their injury.

**Methods:**

We performed a single center retrospective study of burn injured patients admitted to our American Burn Association verified burn center from June 2022 to June 2023. In May 2022, our burn center adopted the American Association for Surgeons in Trauma (AAST) protocol for enoxaparin dosing. This includes twice daily administration of enoxaparin, followed by measurement of anti-Xa levels prior to administration of the fourth or fifth dose. We previously administered a standard dose of 30mg twice daily. Anti-Xa levels were used to guide enoxaparin dose adjustments to remain within prophylactic range. Patients hospitalized for under 48 hours, with glomerular filtration rate less than 30, and with repeated refusal of enoxaparin were excluded.

**Results:**

Eighty seven patients met our inclusion criteria. Fifty six patients started at 40mg twice daily dosing; the remainder started at 30mg twice daily dosing. Of these patients, forty two (48%) required dose adjustments by anti-Xa levels. Of those who required dose adjustment, thirty three (78%) required an increase. There was no significant difference in BMI (p=0.25) or inhalation injury (p=0.62) between patients who did and did not require enoxaparin titration. Patients who required titration had a non-significant trend toward larger percent total body surface area affected (p=0.08). There was also a significantly greater change in dosing for patients who required an increase in dose adjustment as opposed to those who required a decrease in dose adjustment (p < 0.0001). Finally, there was no difference in post-operative bleeding between patients who did and did not require dose titration (p=0.57).

**Conclusions:**

Our results demonstrate that an individual approach to VTE prevention through enoxaparin titration is necessary in order to maintain appropriate prophylactic doses in burn injured patients. Furthermore, adoption of the AAST guidelines is safe and does not result in an increase in post-operative bleeding events.

**Applicability of Research to Practice:**

This work highlights the safety and benefit of enoxaparin titration in burn injured patients to prevent VTE.

